# Rab39a and Rab39b Display Different Intracellular Distribution and Function in Sphingolipids and Phospholipids Transport

**DOI:** 10.3390/ijms20071688

**Published:** 2019-04-04

**Authors:** Julián Gambarte Tudela, Julio Buonfigli, Agustín Luján, Mariano Alonso Bivou, Ignacio Cebrián, Anahí Capmany, María Teresa Damiani

**Affiliations:** 1 Biochemistry and Immunity Laboratory, School of Medicine, University of Cuyo, IMBECU-CONICET, Centro Universitario, Ciudad de Mendoza, Mendoza 5500, Argentina; jgambartetudela@gmail.com (J.G.T.); juliobuonfigli@yahoo.com.ar (J.B.); agustinlujan@hotmail.com (A.L.); malonsobivou@gmail.com (M.A.B.); anahicapmany@hotmail.com (A.C.); 2 Instituto de Histología y Embriología de Mendoza (IHEM, Universidad Nacional de Cuyo, CONICET), Facultad de Ciencias Médicas, Mendoza 5500, Argentina; ignaciocebrian@yahoo.com.ar

**Keywords:** Rab39, sphingolipids, intracellular trafficking, Rab isoforms, small GTPases, Rab proteins, lipid transport, *Chlamydia trachomatis*

## Abstract

Rab GTPases define the identity and destiny of vesicles. Some of these small GTPases present isoforms that are expressed differentially along developmental stages or in a tissue-specific manner, hence comparative analysis is difficult to achieve. Here, we describe the intracellular distribution and function in lipid transport of the poorly characterized Rab39 isoforms using typical cell biology experimental tools and new ones developed in our laboratory. We show that, despite their amino acid sequence similarity, Rab39a and Rab39b display non-overlapping intracellular distribution. Rab39a localizes in the late endocytic pathway, mainly at multivesicular bodies. In contrast, Rab39b distributes in the secretory network, at the endoplasmic reticulum/*cis*-Golgi interface. Therefore, Rab39a controls trafficking of lipids (sphingomyelin and phospholipids) segregated at multivesicular bodies, whereas Rab39b transports sphingolipids biosynthesized at the endoplasmic reticulum-Golgi factory. Interestingly, lyso bis-phosphatidic acid is exclusively transported by Rab39a, indicating that both isoforms do not exert identical functions in lipid transport. Conveniently, the requirement of eukaryotic lipids by the intracellular pathogen *Chlamydia trachomatis* rendered useful for dissecting and distinguishing Rab39a- and Rab39b-controlled trafficking pathways. Our findings provide comparative insights about the different subcellular distribution and function in lipid transport of the two Rab39 isoforms.

## 1. Introduction

Cellular function depends on a dynamic, highly coordinated, intracellular organization. Hence, compartmentalization and distribution of proteins and lipids within cells are of primary importance. Intracellular compartments identity and interaction rely on tightly regulated membrane traffic. Rab proteins are master controllers of vesicular transport. They participate in membrane budding and vesicle formation, vesicle motility along cytoskeleton and membrane fusion at the target compartment through the recruitment of proper effectors. Actually, Rab GTPases function as molecular switches that drive intracellular trafficking pathways by cyclical activation and inactivation of membrane-associated GTP-bound and soluble GDP-bound forms [1]. Rab proteins constitute the most numerous family of the Ras-like small GTPase superfamily. There are more than 60 different Rab proteins; many of them have isoforms with different temporal or tissular expression patterns. Each Rab protein controls a defined transport step along endocytic and exocytic routes [2]. Intracellular traffic complexity substantiates Rab proteins variety and their non-overlapping functions [3]. Notwithstanding the progress on knowledge about vesicular transport, the role of many Rabs and their isoforms remains unclear.

In this work, we aimed to analyze the intracellular distribution and function in lipid transport of the scarcely known Rab39. This protein has two isoforms, Rab39a and Rab39b, which display 76.9% identity at the amino acid (AA) level. On the one hand, Rab39a, which is encoded on chromosome 11, is ubiquitously expressed in human tissues, mainly in epithelial cells. It was first isolated from the cDNA library of human dendritic cells [4]. Initially, it was described as a Golgi-associated protein involved in endocytosis [4]. However, the latest reports point out a different intracellular distribution and functions in phagosome acidification and interleukin secretion [5,6]. On the other hand, Rab39b, present on chromosome Xq28 and predominantly expressed in neuronal cells, was identified in a human fetal brain cDNA library by large-scale sequencing analysis [7]. It is involved in autophagy within neurons, nervous system development and synapse formation. Mutations of Rab39b are related to X-linked mental retardation, autism, Parkinson′s disease and other severe alterations of the nervous system [8,9,10,11]. Recently, it has been shown that Rab39b regulates the transport towards the plasma membrane of GluA2, a subunit of alpha-amino-3-hydroxy-5-methyl-4-isoxazole propionic acid receptors (AMPARs) [12], and the translocation of huntingtin to the endoplasmic reticulum (ER) upon treatment with cAMP analogs [13]. 

At present, intracellular localization and function of both Rab39 isoforms have not been clearly established. This is partly due to Rab39a and Rab39b having differential expression in tissues, which also impedes comparative studies. Some reports localize both isoforms at the Golgi apparatus without distinguishing their particularities [4,14,15]. Here, in addition to the characterization of the intracellular distribution of Rab39 isoforms, we took advantage of using *Chlamydia trachomatis*-infected cells as an experimental model for studying intracellular trafficking, particularly vesicular transport of lipids. 

*Chlamydia trachomatis* (CT) is a worldwide spread obligate intracellular pathogen that inhabits and replicates within a vacuole in host cells [16,17]. This CT-containing vesicle is termed the inclusion. It is a unique vacuole that intercepts several intracellular trafficking pathways to acquire host molecules necessary for bacterial growth. Host lipids are critical for chlamydial replication, thereby CT intercepts lipid-enriched vesicles in transit to the plasma membrane [18,19]. As many other intracellular pathogens, CT seizes intracellular trafficking by hijacking Rab-controlled transport steps [20,21]. It is well known that a subset of Rabs belonging to the recycling and biosynthetic pathways are recruited to chlamydial inclusions. Among them, we described the recruitment of Rab39a [22,23,24,25,26,27]. In this study, using tools developed in the laboratory and CT-infected cells as an experimental model to assess vesicular trafficking, we comparatively described Rab39a and Rab39b intracellular distribution, cargo specificity and function in lipid transport. 

## 2. Results

### 2.1. Rab39a and Rab39b Intracellular Distribution

**Figure 1:**
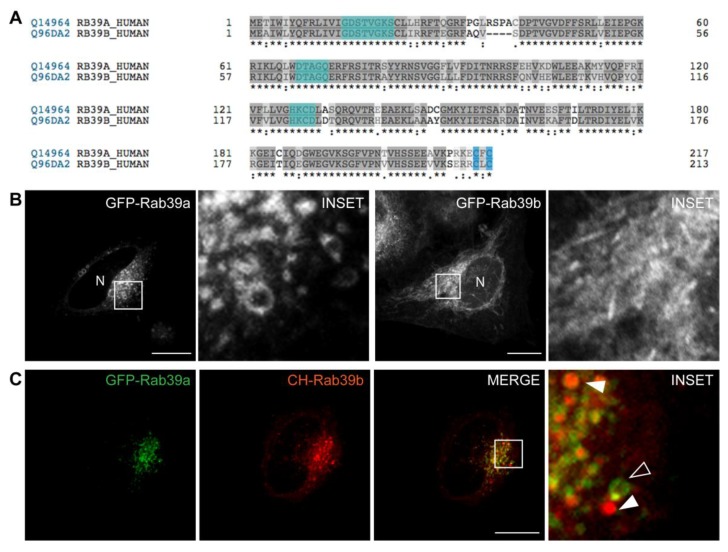
Rab39a and Rab39b isoforms. (**A**) In silico alignment with Uniprot of Rab39a and Rab39b proteins. Amino acid identity of both isoforms is 76.9% (grey) with identical nucleotide binding sites (cyan) and C-terminal prenylation signals (light blue). (**B**) HeLa cells overexpressing GFP-Rab39a (**left**) or GFP-Rab39b (**right**). N indicates nuclei. (**C**) GFP-Rab39a (green) and CH-Rab39b (red) were co-expressed in HeLa cells. Pearson coefficient (r) was calculated with the analysis of 30 cells with ImageJ. The open arrowhead indicates a vesicle decorated with GFP-Rab39a, whereas filled arrowheads point out CH-Rab39b-labeled vesicles. (**B**,**C**) Images were taken by live cell spinning disk microscopy. Images are representative of three independent experiments. Scale bars represent 10 µm.

Rab39 is a small GTPase barely characterized to date. It has two isoforms: Rab39a of 217 AA and Rab39b of only 213 AA. They share 76.9% identity in their AA composition (grey), contain identical nucleotide binding sites (cyan) and the same C-terminal prenylation signals (blue) (Figure 1A). Few reports are published about Rab39 function, and none of them compare similarities and differences between both Rab39 isoforms. Expression in different tissues and the limitations of the commercial and homemade antibodies represent constraints on the characterization of the intracellular distribution of Rab39a and Rab39b. Therefore, we overexpressed both Rab39 isoforms tagged with fluorescent proteins in the same cell type for comparative analysis. As shown in the images, Rab39a displays a punctuate pattern corresponding to a mix of small and big vesicles mainly concentrated at the perinuclear region (Figure 1B, left) whereas Rab39b, in addition to small vesicles at the perinucleus, displays a reticular distribution throughout the entire cytoplasm (Figure 1B, right). Images of live cells were taken by spinning disk to avoid fixation artifacts. Endogenous Rab39a was detected in HeLa cells and displayed the same subcellular distribution as the corresponding overexpressed GFP-protein (Figure S1). In contrast, independently of the cell line tested, there are only two reports showing endogenous Rab39b by immunofluorescence [13,28]. By using the same antibodies, one of them generously provided by Dr. Bruno Goud (Curie Institute, Paris, France), we did not obtain any proper specific staining of endogenous Rab39b that allows the analysis of its intracellular distribution. At present, there are no reports distinguishing Rab39 isoforms’ localization. Thereby, we analyzed the degree of overlapping of GFP-Rab39a (green) and Cherry (CH)-Rab39b (red) in cells overexpressing both proteins. Notwithstanding both isoforms concentrated at the perinuclear area, images suggest that they are not on the same intracellular structures. Indeed, Pearson coefficient (r = 0.5) shows substantial but not total colocalization of the two isoforms, therefore we decided to further characterize Rab39a and Rab39b intracellular distribution (Figure 1C)

**Figure 2:**
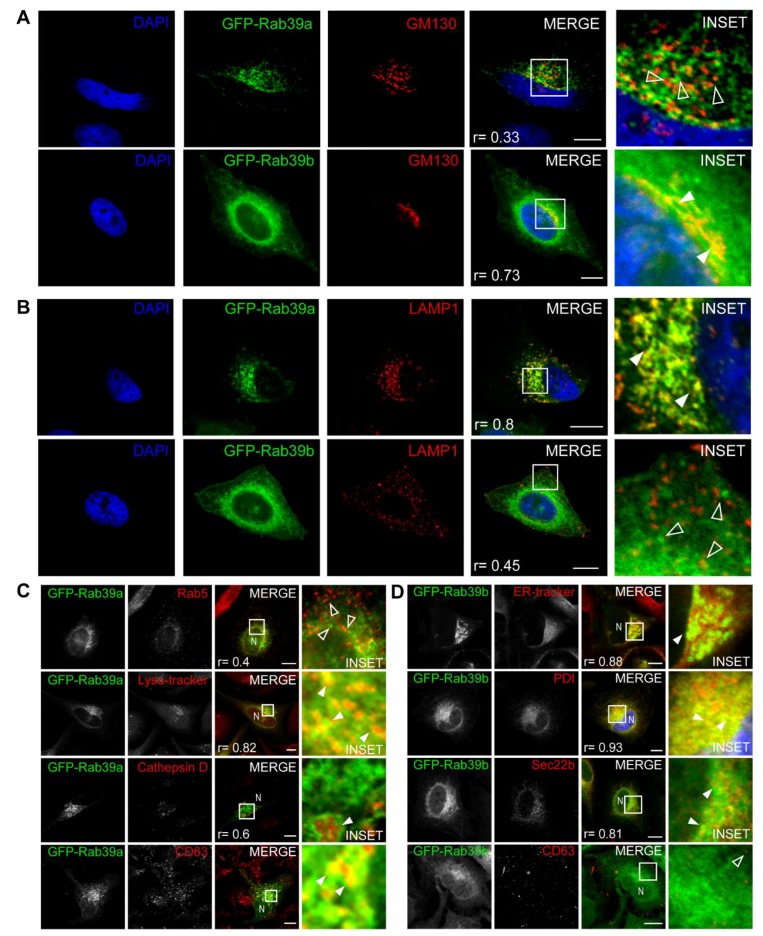
Rab39a and Rab39b intracellular distribution. (**A**,**B**) HeLa cells overexpressing GFP-Rab39a (**top**) or GFP-Rab39b (**bottom**) were incubated with anti-GM130 (**A**) to detect the *cis*-Golgi matrix protein or with anti-LAMP1 (**B**) to detect late endosomes. Cy3-labeled IgG was used as secondary antibody (red). DNA was stained with DAPI (blue). (**C**) Labeling of different intracellular structures of the endocytic pathway with typical probes (lyso-tracker) or specific antibodies (Rab5, Cathepsin D or CD63) followed by the appropriated secondary Cy3-coupled IgG (red) in HeLa cells overexpressing GFP-Rab39a. N indicates nuclei. (**D**) Identification of different intracellular organelles of the secretory pathway with typical probes (ER-tracker) or specific antibodies (PDI, Sec22b or CD63) followed by the appropriated secondary Cy3-coupled IgG (red) in HeLa cells overexpressing GFP-Rab39b. N indicates nuclei. (**A**–**D**) Pearson coefficients (r) were calculated with the analysis of 30 cells with ImageJ for each condition. Open arrowheads show structures bearing a single marker, whereas filled arrowheads indicate colocalization between two labels. Images are representative of three independent experiments. Scale bars represent 10 μm.

Initial reports indicated that both Rab39 isoforms reside at the Golgi apparatus; however, increasing evidence in line with our findings suggest that Rab39a and Rab39b do not share the same intracellular distribution. To solve this controversy, we immunodetected the *cis*-Golgi matrix protein (GM130) in cells overexpressing each GFP-Rab39 isoform. As shown in the images, Rab39a vesicles are in close apposition to the structures labeled with the Golgi marker; however, most of them do not bear both labels (Figure 2A, top, open arrowheads, r = 0.33). In contrast, Rab39b shows overlapping with GM130, indicating that it localizes at the Golgi apparatus (Figure 2A, bottom, filled arrowheads, r = 0.73). Previous findings from our lab prompted us to analyze the distribution of Rab39 isoforms in organelles from the late endocytic compartment such as late endosomes and lysosomes identified with the lysosome-associated membrane protein 1 (LAMP1). Rab39a strongly associated with LAMP1-positive vesicles (Figure 2B, top, filled arrowhead, r = 0.8) compared to Rab39b (Figure 2B, bottom, open arrowheads, r = 0.45). These observations suggest the distribution of Rab39a at the endocytic pathway and Rab39b at the biosynthetic/exocytic via. On the one hand, we performed an exhaustive characterization of Rab39a distribution along the endocytic route by the analysis of its colocalization with early endosomes identified with Rab5 (r = 0.4), late endosomes/lysosomes labeled with lyso-tracker (r = 0.82) or cathepsin D (r = 0.6), and multivesicular bodies (MVBs) pointed out with CD63 (r = 0.85). Our findings depicted in Figure 2C show that Rab39a mainly labels MVBs that are acidic with low degradative enzymatic activity. These organelles are mature late endosomes with intraluminal vesicles called exosomes [22,29,30]. They are strategically located at the intersection of the endocytic and exocytic routes; thus, they receive material internalized by the cells and compounds biosynthesized at the Golgi apparatus. On the other hand, Rab39b distribution along the biosynthetic pathway was confirmed by its high degree of colocalization with markers of the endoplasmic reticulum (ER) such as the ER-tracker (r = 0.88) and phosphodisulfide isomerase (PDI) (r = 0.93), and with Sec22b (r = 0.81) that identifies the ER-Golgi intermediate compartment (ERGIC). As Rab39b strongly colocalized with markers of the early secretory pathway, it could likely regulate trafficking events at the ER-Golgi interface. On the contrary, Rab39b did not significantly colocalize with CD63 (r = 0.41), indicating that this isoform is not present at MVBs (Figure 2D). Pearson coefficients (r) indicate the degree of colocalization between each Rab39 isoform and the organelle markers.

Thus far, our results show that both Rab39 isoforms display different intracellular distribution, being Rab39a mainly distributed at the late endocytic pathway and Rab39b at the early biosynthetic route. Hence, these findings suggest that Rab39 isoforms exert non-overlapping functions and likely control different steps of vesicular transport.

### 2.2. Motility of Rab39a- and Rab39b-Vesicles Along Microtubules

**Figure 3:**
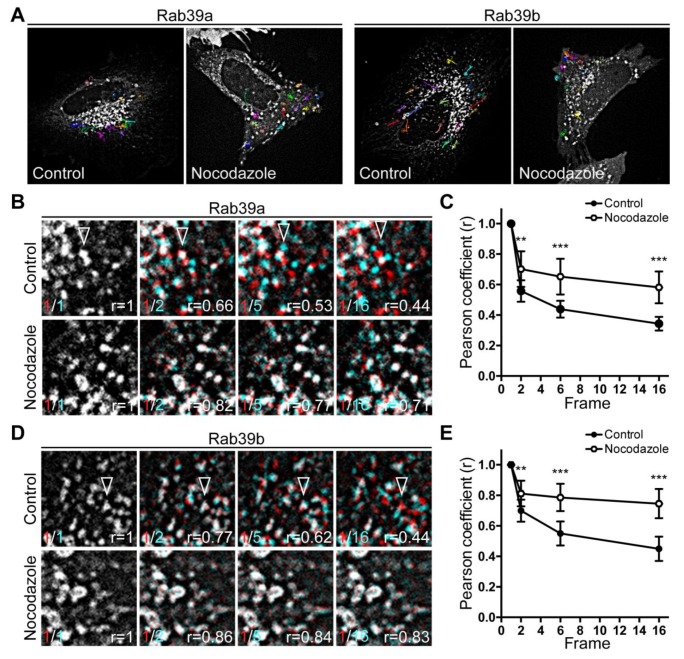
Movement of Rab39a- and Rab39b-vesicles along microtubules. HeLa cells overexpressing GFP-Rab39a or GFP-Rab39b were incubated with DMSO (control) or with 10 μM nocodazole for 1 h at 37 °C. Images were taken every 1 s during 16 s by spinning disk microscopy. (**A**) Trajectories of the selected vesicles in control and nocodazole-treated cells were drawn by MTrackJ FiJi plugin. Images are representative of ten independent experiments. (**B**,**D**) Selected frames of movies of HeLa cells overexpressing GFP-Rab39a (**B**) or GFP-Rab39b (**D**) under control conditions (**top**) and nocodazole treatment (**bottom**). Open arrowheads point out a selected vesicle. r indicates Pearson correlation coefficient between the position of the vesicles in the first frame (red) and their localization in the subsequent frames (cyan). White pixels correspond to the overlapping between the initial and the actual position of the vesicles. (**C**,**E**) Line graphs represent the Pearson correlation coefficients calculated for every vesicle within the whole cell in the experimental conditions shown in (**B**,**D**). Control cells are indicated with filled circles (*n* = 12 for Rab39a and *n* = 10 for Rab39b) and nocodazole-treated cells are represented by empty circles (*n* = 16 for Rab39a and *n* = 8 for Rab39b). Significance was calculated by resampling techniques (** *p* < 0.01 and *** *p* < 0.001).

The cytoskeleton is essential for the spatial organization of intracellular organelles and for the transport of material between them [31,32]. Many molecular components of the cytoskeleton have been described among Rab effector proteins [33,34]; therefore, we aimed to analyze the contribution of microtubules on the movement of Rab39a- and Rab39b-vesicles. Initially, cells overexpressing GFP-Rab39a or GFP-Rab39b were treated with 10 μM nocodazole, a microtubule-disrupting agent, for 1 h at 37 °C before fixation. Live imaging showed the effectiveness of nocodazole in impairing vesicular movement of both Rab39a-labeled (Video S1) and Rab39b-labeled (Video S2) vesicles. We show the trajectories of selected vesicles within untreated and nocodazole-treated cells (Figure 3A). Traditional movement analysis, such as single particle tracking measurements, becomes troublesome when evaluating crowded systems. Thus, we applied the spatiotemporal image correlation spectroscopy (STICS) [35] to measure the displacement of vesicles from their initial position by the determination of the Pearson correlation coefficient (r). Hence, the decrease of correlation between the initial position of vesicles and their subsequent position after a very brief period reflects their displacement. Then, we calculated the Pearson correlation coefficient (r) between the position of the vesicles at the beginning of the measurement (Frame 1) and their position in the successive frames along time (Figure S2). In HeLa cells overexpressing the corresponding GFP-tagged proteins, we analyzed the dynamics of vesicles labeled with Rab39a (Figure 3B,C) or Rab39b (Figure 3D,E) under control conditions or after depolymerization of microtubules by nocodazole treatment. Our results demonstrate that both Rab39a-vesicles and Rab39b-vesicles move along microtubular tracks since vesicular displacement from their initial position decreased in cells in which microtubules were disrupted (Figure 3B,D). Our results show that Pearson correlation coefficients were higher in the presence of nocodazole compared to untreated cells, indicating that both Rab39 isoforms require intact microtubules to transport vesicles. Interestingly, the higher values of Pearson coefficients found during analysis of the movement of Rab39b-labeled vesicles (r = 0.74 ± 0.09 for Rab39b versus r = 0.58 ± 0.1 for Rab39a) indicate that Rab39b appears to be more sensitive to the depolymerization of microtubules (Figure 3C,E). Overall, both Rab39 isoforms control the movement of vesicles along the microtubule cytoskeleton, contributing to the intracellular organelle organization and accurate targeting of vesicular cargos.

### 2.3. Dynamics of Rab39a- and Rab39b-Vesicles

**Figure 4:**
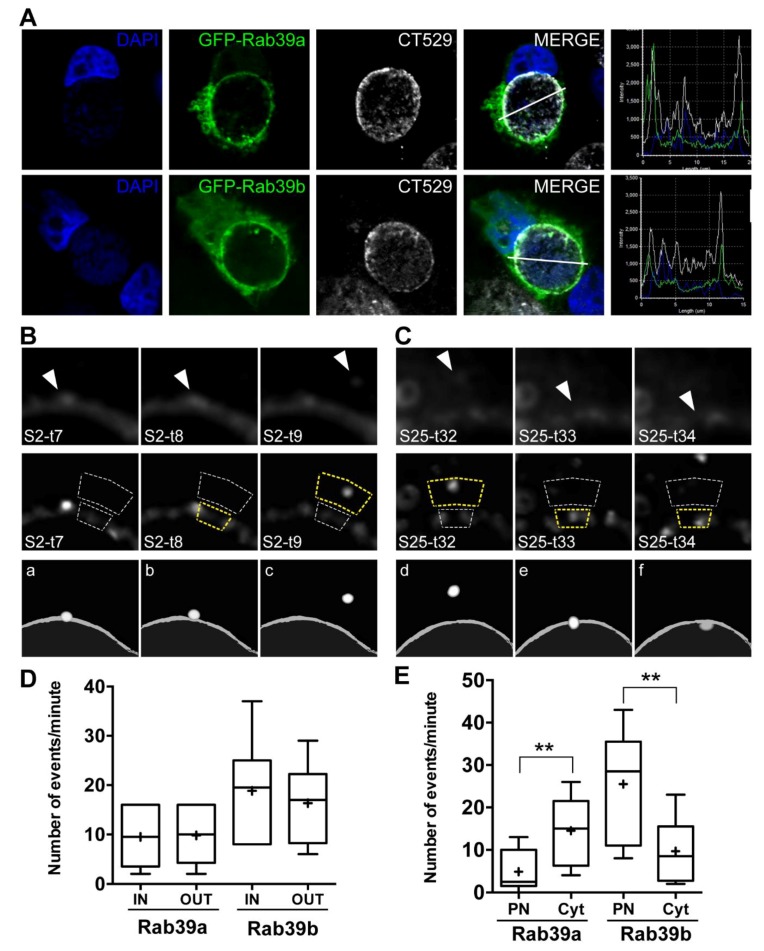
Analysis of the interaction of Rab39a- or Rab39b-labeled vesicles with chlamydial inclusions. HeLa cells overexpressing GFP-Rab39a or GFP-Rab39b were infected with CT (MOI 1) for 24h. (**A**) Rab 39a (**top**) and Rab39b (**bottom**) are shown in green. Bacterial protein CT529 was detected by immunofluorescence (white). DNA was labeled with DAPI (blue). Intensity profiles show Rab39 (green) and CT529 (white) labels across the line that traverses the inclusion (**right**). (**B**,**C**) Images were taken by spinning disk microscopy every 1 s during 60 s: (**top**) sections (S) of the chlamydial inclusion membrane at the indicated times (t); (**middle**) previous images with an enhanced intensity signal of moving object, where dotted line polygons indicate regions of interest (ROIs) and yellow ROIs indicate a peak of intensity; and (**bottom**) schematic representations of previous images that correspond to the departure (a–c) or arrival (d–f) of vesicles to the surroundings of the chlamydial inclusion, respectively. (**D**) Quantification of Rab39-labeled vesicles that reach and leave the chlamydial inclusion area per minute in each confocal plane. (**E**) Inclusions were split into two halves: the perinuclear side (PN) and at the opposite side called cytosol (Cyt). The amount of events that occurred per minute was quantified in both regions. Data correspond to the analysis of chlamydial inclusions from six independent experiments for each Rab39 isoform. Central lines represent medians and crosses depict the means (Bootstrap test, ** *p* < 0.01).

To further delineate the contribution of each Rab39 isoform to the transfer of material between intracellular compartments, we decided to use *Chlamydia trachomatis*-infected cells as an experimental model. CT survives and replicates in a single vesicle, the inclusion, that localizes at the perinucleus. This obligate intracellular pathogen heavily depends on eukaryotic lipids which are obtained by intercepting certain Rab-controlled transport steps. First, we assessed the association of each Rab39 isoform with chlamydial inclusions in HeLa cells overexpressing GFP-Rab39a (Figure 4A, top) or GFP-Rab39b (Figure 4A, bottom). Chlamydial inclusion membrane was evidenced by immuno-labeling the bacterial protein CT529. DAPI stained bacterial and eukaryotic DNA. Intensity profiles show Rab39 (green) and CT529 (white) labels across the line that traverses the indicated inclusion (Figure 4A, right). We confirmed previous findings of our laboratory that indicate that Rab39a [22] and Rab39b (unpublished results) concentrated at the chlamydial inclusion border identified by the bacterial protein CT529. In addition, in CT-infected cells, we determined that endogenous Rab39a almost completely overlaps with GFP-Rab39a-labeled structures as in uninfected cells, indicating that there is not a misplacement of overexpressed GFP-Rab (Figure S3). Furthermore, we analyzed colocalization between different markers of intracellular organelles and GFP-Rab39 isoforms in CT-infected cells. Our results show that chlamydial infection did not alter the distribution of Rab39a at the late endocytic pathway and Rab39b at the early secretory route (Figure S4). 

Our findings show that vesicles labeled with either Rab39a or Rab39b differentially moved to and from the chlamydial inclusion. Therefore, we developed an ImageJ application to analyze the arrival and departure of Rab39a- or Rab39b-vesicles from the surroundings of the inclusion. Video S3 shows the analysis performed at the inclusion borders in cells overexpressing GFP-Rab39a (Video S3, left) or GFP-Rab39b (Video S3, right). The application measures the intensity of the signal in two regions of interest (ROIs). The inclusion membrane is the inner ROI and the adjacent cytosolic area, the outer ROI. The analysis was done in all sections (S) in which the inclusion was split, in images taken every second (t) during 1 min. The increase in signal intensity at the inclusion membrane (inner ROI) counts as an event. We considered that the vesicle goes out of the inclusion membrane area (OUT) when there is an intensity peak in the outer ROI at the immediately following time (Figure 4B, inclusion section 2, from 7 s to 9 s). On the contrary, when the signal intensity peak in the outer ROI takes place in the immediately previous time, we considered that the vesicle enters into the inclusion area (IN) (Figure 4C, inclusion section 25, from 32 s to 34 s). Figure 4B,C (top) shows raw images; Figure 4B,C (middle) corresponds to the respective images with an enhanced intensity signal of moving objects in which dotted line polygons indicate inner and outer ROIs; and t Figure 4B,C (bottom) displays schematic representations of the departure (Figure 4B,C,bottom, a–c) and arrival (Figure 4B,C, bottom, d–f) of vesicles to the inclusion membrane area, respectively. Both Rab39 isoforms actively interacted with the chlamydial inclusion; however, the amount and kind of events that prevailed for each protein were different. Analysis of the results indicated that there was higher interaction between Rab39b-labeled vesicles and the chlamydial inclusion compared to Rab39a-vesicles (Figure 4D). In addition, in Rab39a-overexpressing cells, it was registered a similar amount of vesicles that reach (IN) and depart (OUT) from the inclusion membrane area. In contrast, we observed a slightly but not significantly higher amount of oncoming vesicles in Rab39b-overexpressing cells (Figure 4D). An interesting finding was that Rab39a- and Rab39b-controlled events took place at different sections of chlamydial inclusions. To further analyze this observation, inclusions were divided into two halves: the perinuclear side (PN) and the opposite one that faces the cytosol (Cyt). We found that Rab39a-labeled vesicles tended to interact with chlamydial inclusions at the cytosolic side while the majority of the events registered for Rab39b-labeled vesicles occurred at the PN (Figure 4E).

Altogether, these analyses show that Rab39 isoforms display different vesicular dynamics. Thereby, they likely exert different functions in intracellular transport.

### 2.4. Rab39a- and Rab39b-Mediated Lipid Transport

CT growth and replication rely on scavenging lipids from the host cell by the interception of certain Rab-controlled trafficking pathways. Hence, we took advantage of using CT-infected cells to characterize Rab39-mediated intracellular trafficking and to distinguish the function of Rab39a and Rab39b isoforms in lipid transport. 

**Figure 5:**
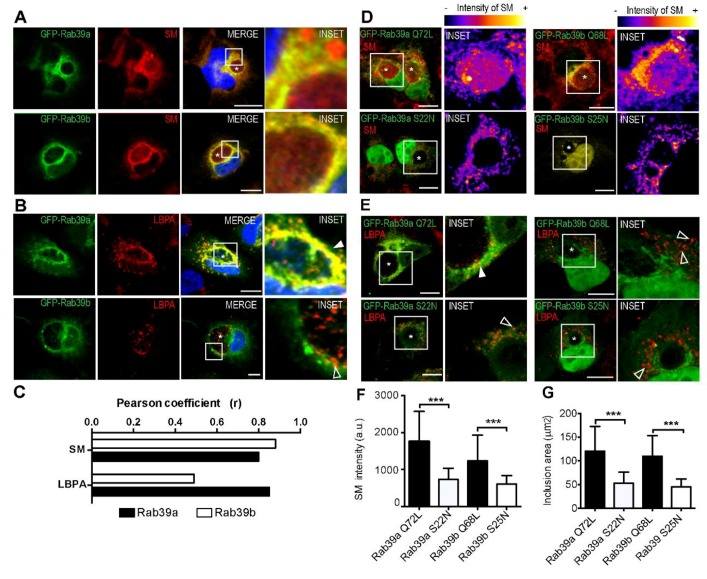
Rab39-controlled lipid transport in CT-infected cells. (**A**–**C**) Cells overexpressing GFP-Rab39aWT (**top**) and Rab39bWT (**bottom**) were infected with CT for 24 h (MOI 1). Asterisks point out chlamydial inclusions. (**A**) Before fixation, cells were incubated with BODIPY-Tr-Ceramide that turns into sphingomyelin (SM) at the Golgi apparatus. (**B**) Cells were fixed and incubated with anti-LBPA antibodies to detect lysobisphosphatidic acid. White arrowhead points out the colocalization between LBPA and Rab39a, and open arrowheads show LBPA-containing vesicle that do not bear Rab39b mark. Images are representative of three independent experiments performed in duplicates. Scale bars represent 10 µm. (**C**) Colocalization index of Rab39 isoforms and SM or LBPA in CT-infected cells. Pearson coefficients (r) were calculated with ImageJ software by the analysis of 30 cells of each experimental condition. (**D**–**G**) Cells overexpressing the positive mutants GFP-Rab39aQ72L or GFP-Rab39bQ68L, or the negative mutants Rab39aS22N or Rab39bS25N were infected with CT for 24 h (MOI 1). Asterisks point out chlamydial inclusions. (**D**) Infected cells were incubated with BODIPY-Tr-Ceramide to label sphingomyelin (SM) or (**E**) anti-LBPA antibodies to detect lysobisphosphatidic acid. Images are representative of two independent experiments. Scale bars represent 10 µm. (**D**) Intensity of fluorescent SM within and at the surroundings of chlamydial inclusions. In color scale, blue indicates the lowest amount of SM whereas yellow corresponds to the highest amount of SM. (**E**) Colocalization between LBPA and Rab39aQ72L is shown with a filled arrowhead, while open arrowheads show LBPA-containing vesicles without Rab39 labeling. (**F**) Bar graph represents the fluorescence intensity of the red labeling (SM) inside of the inclusions quantified by confocal microscopy and expressed as arbitrary units. Data were obtained from two independent experiments (*n* = 30, *** *p* ˂0.001, Student *t* test). (**G**) Bars show the area of chlamydial inclusions, measured by confocal microscopy. Data are representative of two independent experiments (*n* = 30, *** *p* ˂ 0.001, Student *t* test).

First, we investigated the contribution of each Rab39 isoform in the delivery of sphingolipids to the chlamydial inclusion. Both GFP-Rab39a and GFP-Rab39b strongly overlapped with sphingomyelin synthesized from Bodipy-Tr-Ceramide at the Golgi apparatus, as shown by Pearson coefficients (r) greater than 0.8. Rab39 isoforms colocalize with sphingomyelin (SM) not only at the chlamydial inclusion membrane but also in vesicles throughout the cell (Figure 5A). We confirmed these findings in live cells in which Rab39a accumulated in vesicular structures carrying sphingolipids, whereas Rab39b displayed a more reticular pattern (Video S4). To further assess the contribution of Rab39 in SM transport, we overexpressed the positive GTP-bound mutants of both Rab39 isoforms (Rab39a Q72L and Rab39b Q68L), as well as the negative GDP-bound mutants (Rab39a S22N and Rab39b S25N). Then, we infected cells and analyzed the transport of SM towards the chlamydial inclusion. The amount of SM that reached the chlamydial inclusions was substantially higher in cells overexpressing both Rab39a and Rab39b GTP-bound mutants, particularly, Rab39aQ72L (Figure 5D, top). In contrast, little SM was found within inclusions in cells overexpressing the negative mutants of both Rab39 isoforms (Figure 5D, bottom). Fire scale shows the intensity of fluorescent SM. Taken together, these findings indicate that Rab39 is involved in the transport of SM and its delivery to the inclusions. Furthermore, quantification of SM within chlamydial inclusions by confocal microscopy confirmed our observations (Figure 5F). Accordingly, cells overexpressing the positive mutants of Rab39 developed bigger inclusions than those cells that overexpressed the negative mutants (Figure 5G). Altogether, these results indicate that Rab39a and Rab39b participate in the transport of eukaryotic sphingolipids that are required for chlamydial inclusion growth. 

Despite the strong colocalization between both Rab39 isoforms and sphingomyelin, images suggest that they do not exert identical function in lipid transport. Hence, we decided to analyze Rab39 isoforms function in the transport of phospholipids. Interestingly, GFP-Rab39a almost completely colocalized with lyso bis-phosphatidic acid (LBPA) (Figure 5B, top, filled arrowhead). In contrast, we observed little colocalization between this phospholipid and the other isoform, GFP-Rab39b. It could be appreciated that the small vesicles carrying LBPA that are within chlamydial inclusions are devoid of Rab39b (Figure 5B, bottom, open arrowheads). These findings are in agreement with our observations about the differential intracellular distribution of both Rab39 isoforms. Actually, MVBs are late endocytic organelles enriched in LBPA; therefore, it is likely that Rab39a, but not Rab39b, drives the transport of phospholipids. On the one hand, in cells overexpressing Rab39aQ72L, we appreciated small LBPA-containing vesicles within chlamydial inclusions decorated by the Rab protein (Figure 5E, top left, filled arrowhead). On the contrary, in cells overexpressing the negative Rab39aS22N mutant, most LBPA-labeled vesicles were in the surroundings, but not inside the inclusion (Figure 5E, bottom left, open arrowhead). On the other hand, in cells overexpressing either GTP-bound or GDP-bound mutants of Rab39b, the lack of colocalization with LBPA, confirmed that Rab39b isoform does not participate in the transport of phospholipids (Figure 5E, right). Overall, our results indicate that colocalization with sphingomyelin or phospholipids depends on the intracellular distribution of each Rab39 isoform. 

**Figure 6:**
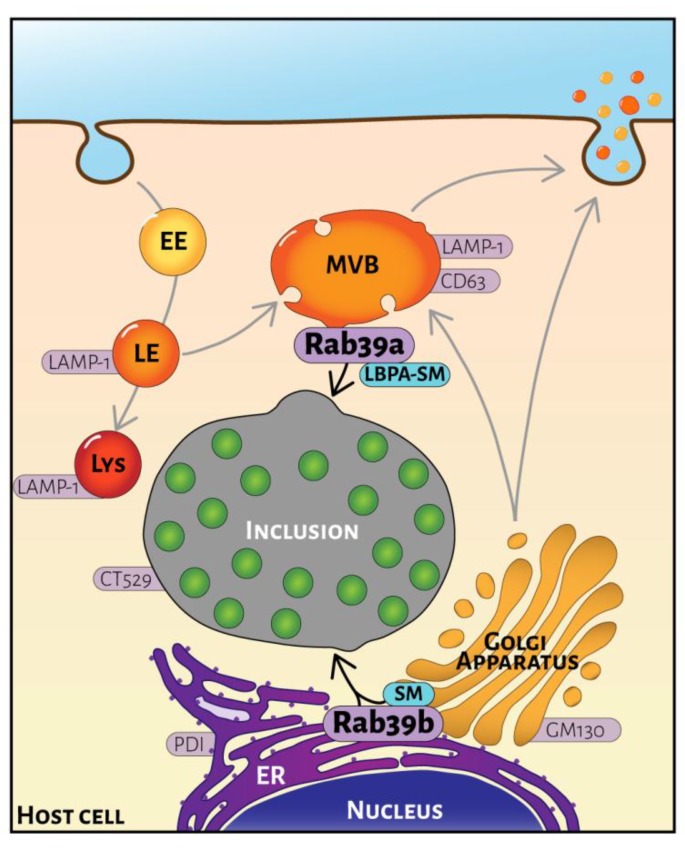
Scheme of Rab39a and Rab39b intracellular distribution in *Chlamydia trachomatis*-infected cell. Rab39a localizes at the late endocytic pathway mainly at multivesicular bodies (MBVs) labeled with CD63 and LAMP1. Rab39a transports sphingolipids (SM) and phospholipids (LBPA) from MBVs to the chlamydial inclusion. In contrast, Rab39b distributes at the early secretory route, particularly at the interface between the endoplasmic reticulum (ER) and the *cis*-Golgi. PDI labels the ER and GM130 identifies the Golgi apparatus. Rab39b controls the delivery to the chlamydial inclusion of newly synthesized sphingolipids (SM). EE, early endosomes; LE, late endosomes; Lys, Lysosomes.

In summary, our findings indicate that Rab39a and Rab39b have different non-overlapping functions in lipid transport to the chlamydial inclusion. In uninfected cells, it is more difficult to evaluate the role of Rab isoforms in lipid transport. For this reason, we used CT-infected cells as a tool for the assessment of the function on intracellular traffic of proteins that interact with inclusions, particularly the analysis and comparison of different Rab isoforms. The use of CT-infected cells facilitates the visualization that Rab39a and Rab39b control lipid transport from different intracellular sources. Although both isoforms participate in sphingomyelin trafficking, either from MVBs (Rab39a) or the ER-Golgi factory (Rab39b); the lack of colocalization between Rab39b and LBPA supports the concept that these proteins do not exert the same function on phospholipid transport. A schematic representation is depicted in Figure 6, which summarizes the intracellular distribution of Rab39a and Rab39b and the main intracellular transport steps controlled by each Rab39 isoform in CT-infected cells.

## 3. Discussion

In the past twenty years, the understanding of how Rab proteins control intracellular transport has exponentially increased. Novel genetic and cell biology tools combined with improved biochemical and biophysical approaches have been of valuable help for dissecting individual Rab function. However, knowledge about Rab closely related proteins, commonly known as Rab isoforms, is still limited. 

In this study, we show that despite amino acid sequence similarity, Rab39a and Rab39b display differential subcellular distribution. Rab39a mainly localizes in organelles of the late endocytic pathway such as MBVs and late endosomes, labeled with CD63 and LAMP1. In contrast, Rab39b concentrates in the early secretory route, particularly at the ER-Golgi interface where it extensively colocalizes with PDI, Sec22b, and GM130. Similarly, Rab11 isoforms distribute in different vesicular compartments. In polarized cells, Rab11a and Rab11b do not colocalize on apical recycling endosomes [36]. However, both Rab11 isoforms share effectors and converge in the control of certain trafficking steps of the recycling pathway. Moreover, Rab5 isoforms exert different roles on epidermal growth factor receptor (EGFR) transport, even when they share more than 90% amino acid sequence. Rab5a drives EGFR progression in the endocytic pathway for its degradation, whereas Rab5c does not participate in EGFR transport [37,38]. 

Analysis of Rab27 isoforms is another example of non-overlapping functions in intracellular traffic. Rab27a and Rab27b have 71% amino acid identity and share eleven effectors. However, they exert different roles in the transport of lysosome-related organelles such as melanosomes, azurophilic granules, secretory granules, and platelet dense granules, as well as in the biogenesis and release of multivesicular bodies (MVBs) and exosomes. For instance, MVBs size increases by Rab27a silencing, whereas these organelles redistribute towards the perinuclear region upon Rab27b depletion. In fact, the different effects of individual knockdown of Rab27a or Rab27b GTPases in the exosomal pathway obey differential effector engagement. While Rab27b regulates MVB/exosome docking at the plasma membrane through Slac2-b interaction, Rab27a bound to Slp4 controls exosome secretion [39,40].

Rab proteins rule directional movement of vesicular cargos along microtubule or actin tracks through the association with molecular motors. Until now, there were no studies regarding the contribution of the cytoskeleton in Rab39-mediated intracellular trafficking. Here, we show that both Rab39 isoforms transport vesicles on microtubules, since movement is impaired after nocodazole treatment, indicating the requirement of cytoskeleton integrity for Rab39 activity. In vitro, it has been shown the interaction between Rab39b and myosin Va [28]. However, our results indicate that the movement of Rab39b-vesicles is more affected than Rab39a-vesicles after microtubule depolymerization. 

Many important lessons come from the analysis of how intracellular pathogens subvert vesicular transport. Rab8a and Rab8b functions in membrane trafficking were described by the study of West Nile viral particles transport. In infected cells, viruses are exclusively released by the basolateral side through a Rab8b-controlled route without the participation of Rab8a. Thus, Rab8a rules apical traffic, while Rab8b controls vesicular movement in basolateral transport [41]. 

*Chlamydia trachomatis*, as other intracellular pathogens, has developed effective strategies for the acquisition of host molecules from different intracellular compartments [18,42,43]. Inclusions are strategically placed for the interception of vesicles in transit to or from the plasma membrane. Here, we took advantage of chlamydial requirement for eukaryotic lipids to distinguish different dynamics of vesicles decorated by each Rab39 isoform, indistinguishable in uninfected cells. Interfering with Rab39 function, for instance with GDP-bound mutants, impairs the delivery of eukaryotic sphingolipids to chlamydial inclusions. Consequently, inclusion growth and development are impaired. Notwithstanding Rab39a and Rab39b transport sphingomyelin, Rab39a controls its transport from late endocytic organelles such as MVBs and Rab39b from the ER-Golgi biosynthetic factory.

An interesting finding is that the phospholipid LBPA is exclusively transported by Rab39a, indicating that both isoforms exert non-overlapping roles in intracellular transport. In summary, Rab39b drives the transport at the early secretory pathway of endogenously biosynthesized sphingolipids, whereas Rab39a carries sphingolipids and phospholipids segregated at MVBs. 

Interestingly, vesicular movement in the surroundings of the chlamydial inclusion prevails for Rab39b. We also found greater activity on different areas of the inclusion for Rab39a and Rab39b, supporting the notion of their differential distribution and function. Consistent with Rab39a localization at the MVBs, Rab39a-labeled vesicles predominantly interact with the chlamydial inclusion at the side that faces cytosol. In contrast, the interaction with inclusions of Rab 39b-vesicles mainly occurs at the perinuclear side, in agreement with Rab39b distribution at the early secretory pathway, particularly the ER/*cis*-Golgi interface.

We assume that the complementary but different roles played by Rab39 isoforms in lipid transport in CT-infected cells are due to the interaction with particular yet unknown effectors. Most efforts should be focused in unveiling Rab39 effectors to understand the function of each isoform in intracellular transport at the molecular level. Nevertheless, using CT-infected cells, we contributed to the assessment of Rab39 isoforms functions in intracellular trafficking.

In the present study, we accomplished two achievements: first, we demonstrated the non-overlapping roles of the two Rab39 isoforms in lipid transport, and, second, we showed the convenience of the CT-infected cell model to provide insights into the intracellular transport pathways. 

## 4. Materials and Methods

### 4.1. Cells and Bacteria

HeLa 229 cells (ABAC, Buenos Aires, Argentina) were cultured in high glucose Dulbecco’s modified Eagle’s medium (GIBCO BRL, Buenos Aires, Argentina) supplemented with 10% (*v*/*v*) fetal bovine serum (Internegocios SA, Buenos Aires, Argentina), 0.3 mg/mL L-glutamine (ICN Biomedicals Inc.), and 1.55 mg/mL glucose (Biopack) without antibiotics in 5% CO_2_ at 37 °C. *Chlamydia trachomatis* serotype L2 (CT) were gently given by Unidad de Estudios de *Chlamydia* (Facultad de Farmacia y Bioquímica, Universidad de Buenos Aires, Argentina. For infection experiments, cell and bacteria were centrifuged 15 min at 1000× *g*. After 2 h, the medium was refreshed and cells were cultured for the indicated post-infection times (pi). Bacteria were used at a multiplicity of infection (MOI) of 1.

### 4.2. Antibodies and Reagents

The primary antibodies were: mouse monoclonal anti-GM130 and anti-LAMP1 from BD Biosciences (MI, USA); anti-tubulin from Abcam (MI, USA); anti-CD63 from Zymed (Invitrogen, Buenos Aires, Argentina); anti-LBPA from Merck (Buenos Aires, Argentina); mouse monoclonal anti-Sec22b from Santa Cruz (Buenos Aires, Argentina); anti-PDI from AllScience (MI, USA); anti-Rab5 and anti-cathepsin D from Aviva systems biology (MI, USA), and rabbit polyclonal anti-Rab39 antibody made at Bruno Goud´s laboratory (Curie Institute, Paris, France). Bacteria were detected with anti-CT529 generously provided by Agathe Subtil (Pasteur Institute, Paris, France). The secondary antibodies were: goat Cy3.5-labeled anti-rabbit IgG and goat Cy3.5-labeled anti-mouse IgG from Abcam (MI, USA). Lyso-tracker Red DND-99 and DAPI were from Molecular Probes (Life Technologies, Buenos Aires, Argentina); nocodazole and Mowiol 4-88 were from Calbiochem (San Diego, CA, USA), Bafilomicyn A from Abcam (MI, USA) and Brefeldin A (BFA) and dimethyl sulfoxide (DMSO) from Merck (Buenos Aires, Argentina).

### 4.3. Plasmids and Cell Transfection 

pcDNA.DEST47-GFP-Rab39a WT, pcDNA.DEST47-GFP-Rab39a Q72L, pcDNA.DEST47-GFP-Rab39a S22N, pcDNA.DEST47-GFP-Rab39b WT, pcDNA.DEST47-GFP-Rab39 Q68L and pcDNA.DEST47-GFP-Rab39b S25N plasmids were kindly given by Bruno Goud (Curie Institute, Paris, France). HeLa cells were grown on 12 mm diameter glass coverslips in 24 well plates for 24 h. Cells were washed once with PBS and transfected using XtremeGene 9 (Roche Applied Science, Indianapolis, IN, USA) and Lipofectamine 2000 (Invitrogen, Buenos Aires, Argentina) using 1 μL per 1 µg of DNA per well according to the manufacturer’s instructions. After transfection, cells were incubated with Opti-MEM medium (Invitrogen, Buenos Aires, Argentina). 

### 4.4. Immunofluorescence and Confocal Microscopy

HeLa cells overexpressing GFP-Rab39a or GFP-Rab39b were grown on 12 mm diameter glass coverslips. Cells were fixed in 3% paraformaldehyde (PFA) for 10 min, quenched with 0.05 M NH_4_Cl (20 min), permeabilized in 0.2% saponin and 1 mg/mL BSA (Invitrogen, Buenos Aires, Argentina) in PBS (15 min), and incubated with the appropriated primary and secondary antibodies (60 min). Coverslips were mounted with Mowiol 4-88 containing 0.5 μg/mL DAPI (Molecular Probes, Life Technologies, CA, USA) and examined under an Olympus FV-1000 spectral confocal unit on an IX-25 Olympus inverted microscope with FV10-ASW 1.7 software (Olympus, NY, USA). Adobe CS6 software. 

### 4.5. Time-Lapse Video Microscopy

HeLa cells overexpressing GFP-Rab39a or GFP-Rab39b were grown on 35 mm diameter glass coverslips (Asahi Techno Glass, Tokyo, Japan). The cells were treated with 10 µM nocodazole or DMSO 1 h before the image acquisition. Time-lapse imaging was performed at 37 °C on a spinning disk confocal microscope mounted on an inverted motorized microscope DMIRE2 (Leica, Wetzlar, Germany) equipped with a CSUXI spinning disk head (Yokogawa, Tokyo, Japan) and temperature and CO_2_ controller. Images were captured on a QuantEM 512SC camera (Photometrics Company, AZ, USA). A z stack of three planes was acquired every 1 s for a period of 1 min. Movies were generated using MetaMorph software (San Jose, CA, USA). 

### 4.6. Vesicular Movement Analysis

Individual vesicles were manually tracked using the application MTrackJ for Fiji for the analysis of their trajectories [44]. A temporal image correlation spectroscopy analysis was performed [35]. Movies were normalized and filtered with an unsharp mask (radius = 3, mask weight = 0.8). Pearson correlation coefficient was calculated between the first frame and the successive ones for the same vesicles in ROIs (region of interest) delimited by the cell dimensions. The coefficient obtained for the correlation between frames at different points of time, is used as an indicator of the degree of displacement of marked vesicles. The lower is the coefficient obtained, the greater is the displacement of the vesicles from their initial location. 

An application to quantify the arrival and departure of vesicles from the chlamydial inclusion area was developed as a Fiji macro. Movies generated as mentioned under the subtitle “Time-lapse video microscopy” were background subtracted and cropped at the chlamydial inclusion area. Other treatments were programmed in the macro-like normalization and a filter that enhanced the intensity of moving objects. Then, the application split the chlamydial inclusion in a finite amount of sections (S), the sizes of which were set according to the dimension of the vesicles. In each section, means and maximum intensities values were read in two different regions of interests (ROIs): one over the chlamydial inclusion membrane (inner ROI) and the other right outside the membrane edge (outer ROI). A peak of signal intensity at the inner ROI (inclusion membrane) was recognized as an event. When an intensity peak was found in the outer ROI in the subsequent time (next frame), it meant that a vesicle departed from the chlamydial inclusion surroundings. Conversely, if an intensity peak in the outer ROI was found in the immediately previous time, it was considered that a vesicle arrived to the chlamydial inclusion surroundings. Absence of intensity peaks in outer ROIs were labeled as “not rated” events. All events were tagged with the frame and ROI where they occurred to be subsequently visually checked.

### 4.7. Image Analysis

Confocal images from experiments were analyzed with Image J program and imported into Adobe Photoshop CS6 software. Pearson colocalization coefficient was calculated using JACOP plugging. Intensity profiles were performed and analyzed with FV10 software.

### 4.8. Staining of Lipids and Organelles

HeLa cells overexpressing GFP-Rab39a or GFP-Rab39b and their mutants infected with *Chlamydia trachomatis* strain L2 were incubated with different fluorescent probes before or after cell fixation. Staining of sphingolipids was performed by incubation with 5 µM BODIPY-Tr-Ceramide in complex with BSA (Invitrogen, Buenos Aires, Argentina) at 4 °C for 30 min and then washed with cold PBS to eliminate extracellular probe. Finally, cells were incubated in D-MEM at 37 °C for 45 min before fixation. Endoplasmic reticulum staining in live cells was performed with 1 µM ER tracker following the manufacturer´s protocol (Invitrogen, Buenos Aires, Argentina). Lyso-tracker Red DND-99 was added to live cell cultures. The cells were incubated for 10 min and after washed with PBS three times before fixation.

### 4.9. Statistical Analysis

Statistical analysis was performed using GraphPad Prism 6 software (CA, USA). The data represent the mean ± SEM of *n* experiments. For simple unpaired analysis between two groups, Student’s *t* test was chosen. Data from videos were analyzed by bootstrap test. *p* values less than 0.01 were considered statistically significant.

## Supplementary Materials

Supplementary materials can be found at https://zenodo.org/record/2628327#.XKXgYcQRWUl.

## References

[1] Stenmark H. (2009). Rab GTPases as coordinators of vesicle traffic. Nat. Rev. Mol. Cell Biol..

[2] Zerial M., McBride H. (2001). Rab proteins as membrane organizers. Nat. Rev. Mol. Cell Biol..

[3] Wandinger-Ness A., Zerial M. (2014). Rab proteins and the compartmentalization of the endosomal system. Cold Spring Harb. Perspect. Biol..

[4] Chen T., Han Y., Yang M., Zhang W., Li N., Wan T., Guo J., Cao X. (2003). Rab39, a novel Golgi-associated Rab GTPase from human dendritic cells involved in cellular endocytosis. Biochem. Biophys. Res. Commun..

[5] Seto S., Tsujimura K., Koide Y. (2011). Rab GTPases regulating phagosome maturation are differentially recruited to mycobacterial phagosomes. Traffic.

[6] Becker C.E., Creagh E.M., O’Neill L.A. (2009). Rab39a binds caspase-1 and is required for caspase-1-dependent interleukin-1beta secretion. J. Biol. Chem..

[7] Cheng H., Ma Y., Ni X., Jiang M., Guo L., Ying K., Xie Y., Mao Y. (2002). Isolation and characterization of a human novel RAB (RAB39B) gene. Cytogenet. Genome Res..

[8] Yuan L., Deng X., Song Z., Yang Z., Ni B., Chen Y., Deng H. (2015). Genetic analysis of the RAB39B gene in Chinese Han patients with Parkinson’s disease. Neurobiol. Aging.

[9] Vanmarsenille L., Giannandrea M., Fieremans N., Verbeeck J., Belet S., Raynaud M., Vogels A., Männik K., Õunap K., Jacqueline V. (2014). Increased dosage of RAB39B affects neuronal development and could explain the cognitive impairment in male patients with distal Xq28 copy number gains. Hum. Mutat..

[10] Wilson G.R., Sim J.C.H., McLean C., Giannandrea M., Galea C.A., Riseley J.R., Stephenson S.E.M., Fitzpatrick E., Haas S.A., Pope K. (2014). Mutations in RAB39B cause X-linked intellectual disability and early-onset Parkinson disease with α-synuclein pathology. Am. J. Hum. Genet..

[11] Corbier C., Sellier C. (2017). C9ORF72 is a GDP/GTP exchange factor for Rab8 and Rab39 and regulates autophagy. Small GTPases.

[12] Mignogna M.L., Giannandrea M., Gurgone A., Fanelli F., Raimondi F., Mapelli L., Bassani S., Fang H., Van Anken E., Alessio M. (2015). The intellectual disability protein RAB39B selectively regulates GluA2 trafficking to determine synaptic AMPAR composition. Nat. Commun..

[13] Yao Y., Cui X., Al-Ramahi I., Sun X., Li B., Hou J., Difiglia M., Palacino J., Wu Z.-Y., Ma L. (2015). A striatal-enriched intronic GPCR modulates huntingtin levels and toxicity. eLife.

[14] Yoshimura S., Gerondopoulos A., Linford A., Rigden D.J., Barr F.A. (2010). Family-wide characterization of the DENN domain Rab GDP-GTP exchange factors. J. Cell Biol..

[15] Proikas-Cezanne T., Gaugel A., Frickey T., Nordheim A. (2006). Rab14 is part of the early endosomal clathrin-coated TGN microdomain. FEBS Lett..

[16] Mueller K.E., Plano G.V., Fields K.A. (2014). New frontiers in type III secretion biology: The Chlamydia perspective. Infect. Immun..

[17] Elwell C., Mirrashidi K., Engel J. (2016). Chlamydia cell biology and pathogenesis. Nat. Rev. Microbiol..

[18] Elwell C.A., Engel J.N. (2012). Lipid acquisition by intracellular Chlamydiae. Cell. Microbiol..

[19] Saka H.A., Valdivia R.H. (2010). Acquisition of nutrients by Chlamydiae: Unique challenges of living in an intracellular compartment. Curr. Opin. Microbiol..

[20] Damiani M.T., Gambarte Tudela J., Capmany A. (2014). Targeting eukaryotic Rab proteins: A smart strategy for chlamydial survival and replication. Cell. Microbiol..

[21] Hackstadt T. (2000). Redirection of host vesicle trafficking pathways by intracellular parasites. Traffic.

[22] Gambarte Tudela J., Capmany A., Romao M., Quintero C., Miserey-Lenkei S., Raposo G., Goud B., Damiani M.T. (2015). The late endocytic Rab39a GTPase regulates the interaction between multivesicular bodies and chlamydial inclusions. J. Cell Sci..

[23] Rzomp K.A., Moorhead A.R., Scidmore M.A. (2006). The GTPase Rab4 interacts with Chlamydia trachomatis inclusion membrane protein CT229. Infect. Immun..

[24] Leiva N., Capmany A., Damiani M.T. (2013). Rab11-family of interacting protein 2 associates with chlamydial inclusions through its Rab-binding domain and promotes bacterial multiplication. Cell. Microbiol..

[25] Rzomp K.A., Scholtes L.D., Briggs B.J., Whittaker G.R., Scidmore M.A. (2003). Rab GTPases are recruited to chlamydial inclusions in both a species-dependent and species-independent manner. Infect. Immun..

[26] Rejman Lipinski A., Heymann J., Meissner C., Karlas A., Brinkmann V., Meyer T.F., Heuer D. (2009). Rab6 and Rab11 regulate Chlamydia trachomatis development and golgin-84-dependent Golgi fragmentation. PLoS Pathog..

[27] Capmany A., Damiani M.T. (2010). Chlamydia trachomatis intercepts Golgi-derived sphingolipids through a Rab14-mediated transport required for bacterial development and replication. PLoS ONE.

[28] Lindsay A.J., Jollivet F., Horgan C.P., Khan A.R., Raposo G., McCaffrey M.W., Goud B. (2013). Identification and characterization of multiple novel Rab–myosin Va interactions. Mol. Biol. Cell.

[29] Piper R.C., Katzmann D.J. (2007). Biogenesis and Function of Multivesicular Bodies. Annu. Rev. Cell Dev. Biol..

[30] van Niel G., Porto-Carreiro I., Simoes S., Raposo G. (2006). Exosomes: A Common Pathway for a Specialized Function. J. Biochem..

[31] Kjos I., Vestre K., Guadagno N.A., Borg Distefano M., Progida C. (2018). Rab and Arf proteins at the crossroad between membrane transport and cytoskeleton dynamics. Biochim. Biophys. Acta Mol. Cell Res..

[32] Hunt S.D., Stephens D.J. (2011). The role of motor proteins in endosomal sorting. Biochem. Soc. Trans..

[33] Delevoye C., Goud B. (2015). Rab GTPases and kinesin motors in endosomal trafficking. Methods Cell Biol..

[34] Horgan C.P., McCaffrey M.W. (2011). Rab GTPases and microtubule motors. Biochem. Soc. Trans..

[35] Hebert B., Costantino S., Wiseman P.W. (2005). Spatiotemporal Image Correlation Spectroscopy (STICS) Theory, Verification, and Application to Protein Velocity Mapping in Living CHO Cells. Biophys. J..

[36] Lapierre L.A., Dorn M.C., Zimmerman C.F., Navarre J., Burnette J.O., Goldenring J.R. (2003). Rab11b resides in a vesicular compartment distinct from Rab11a in parietal cells and other epithelial cells. Exp. Cell Res..

[37] Bucci C., Lütcke A., Steele-Mortimer O., Olkkonen V.M., Dupree P., Chiariello M., Bruni C.B., Simons K., Zerial M. (1995). Co-operative regulation of endocytosis by three Rab5 isoforms. FEBS Lett..

[38] Chen P.-I., Kong C., Su X., Stahl P.D. (2009). Rab5 isoforms differentially regulate the trafficking and degradation of epidermal growth factor receptors. J. Biol. Chem..

[39] Fukuda M. (2013). Rab27 effectors, pleiotropic regulators in secretory pathways. Traffic.

[40] Ostrowski M., Carmo N.B., Krumeich S., Fanget I., Raposo G., Savina A., Moita C.F., Schauer K., Hume A.N., Freitas R.P. (2010). Rab27a and Rab27b control different steps of the exosome secretion pathway. Nat. Cell Biol..

[41] Kobayashi S., Suzuki T., Kawaguchi A., Phongphaew W., Yoshii K., Iwano T., Harada A., Kariwa H., Orba Y., Sawa H. (2016). Rab8b Regulates Transport of West Nile Virus Particles from Recycling Endosomes. J. Biol. Chem..

[42] Carabeo R.A., Mead D.J., Hackstadt T. (2003). Golgi-dependent transport of cholesterol to the Chlamydia trachomatis inclusion. Proc. Natl. Acad. Sci. USA.

[43] ZL C., Kumar Y., Fischer E.R., Hackstadt T., Valdivia R.H. (2008). Cytoplasmic lipid droplets are translocated into the lumen of the Chlamydia trachomatis parasitophorous vacuole. Proc. Natl. Acad. Sci. USA.

[44] Meijering E., Dzyubachyk O., Smal I. (2012). Methods for Cell and Particle Tracking. Methods Enzymol..

